# Post-Cholecystectomy nutritional management: a comprehensive review of evidence-based guidelines and clinical practice

**DOI:** 10.3389/fsurg.2026.1911177

**Published:** 2026-08-26

**Authors:** Chuang Guo, Yake Lan, Yu Zhang, Yuan Guo, Chunyan Sun, Minghui Li

**Affiliations:** 1 First Affiliated Hospital of Heilongjiang University of Chinese Medicine, Harbin, Heilongjiang, China; 2 The First Psychiatric Hospital of Harbin, Harbin, Heilongjiang, China; 3 Dalian University Affiliated Xinhua Hospital, Dalian, Liaoning, China; 4 The First Hospital of Harbin, Harbin, Heilongjiang, China

**Keywords:** cholecystectomy, enhanced recovery after surgery, individualized nutritional support, nutritional care, postoperative nutritional management

## Abstract

**Background:**

Cholecystectomy is one of the most common abdominal surgeries worldwide, yet postoperative nutritional management remains variable. This review synthesizes current evidence on nutritional care after cholecystectomy, with a focus on actionable insights for clinicians.

**Methods:**

We conducted a non-systematic narrative review of English-language literature published from January 2010 through June 2026, supplemented by manual searches of guidelines. Due to the narrative design, we did not perform a formal risk-of-bias assessment or meta-analysis.

**Findings:**

Postoperative gastrointestinal symptoms (e.g., bloating, diarrhea, dyspepsia) are common, but their incidence and etiology vary widely. Early oral feeding within 24 h may reduce hospital stay. High-risk patients (diabetes, obesity, elderly) may benefit from protein supplementation (1.2–1.5 g/kg/day) and selective fat-soluble vitamin monitoring when clinically indicated. We provide a risk-stratified algorithm, critically appraise conflicting evidence, and identify knowledge gaps. While standardized ERAS protocols exist for elective cases, complicated scenarios (acute cholecystitis, post-pancreatitis) require special consideration.

**Conclusion:**

This review provides an evidence-informed, cautious framework for individualized postoperative nutritional support, emphasizing that most recommendations are extrapolated from other surgical populations and require validation in cholecystectomy-specific trials.

## 1 Introduction

Cholecystectomy, especially laparoscopic cholecystectomy, is the standard treatment for symptomatic gallstones (1–5). The widespread adoption of minimally invasive techniques has significantly improved perioperative outcomes, reducing hospital stays and postoperative complications (6–8). However,despite these advances,patients undergoing cholecystectomy still face challenges related to postoperative recovery, especially concerning nutritional status and gastrointestinal function (9–12). The surgical removal of the gallbladder alters bile secretion and digestive processes, which combined with surgical stress and transient digestive dysfunction, can profoundly impact patients’ nutritional metabolism (1, 13, 14). These changes may increase the risk of postoperative malnutrition, associated with delayed healing, infection, and longer hospitalization (15, 16). Therefore, optimizing nutritional care is critical to enhance recovery.

The Enhanced Recovery After Surgery (ERAS) concept has revolutionized perioperative care (17). Nutritional management is a cornerstone of ERAS, emphasizing early oral feedingand individualized support (18–20). In cholecystectomy, ERAS has demonstrated benefits (21). However, specific challenges — altered bile flow,fat malabsorption,and post-cholecystectomy diarrhea necessitates tailored strategies (14, 15, 22).

**Figure 1:**
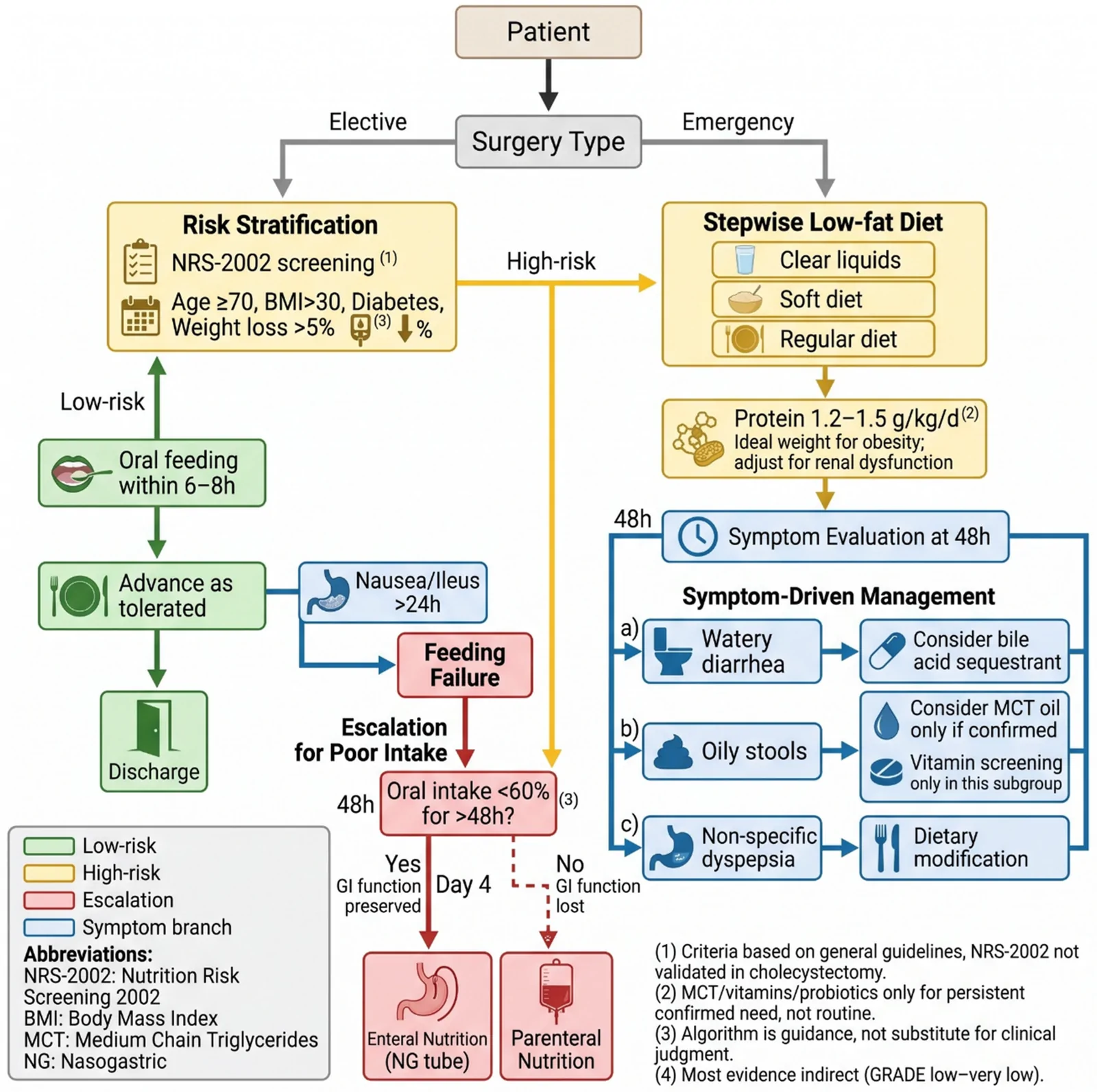
Risk-stratified clinical decision algorithm for postoperative nutritional management after cholecystectomy.

This review aims to provide a critical, evidence-based synthesis of postoperative nutritional management. It addresses: (1) pathophysiological changes; (2) nutritional risk assessment; (3) phase-specific support; (4) special populations and complicated scenarios; and (5) future research. We explicitly distinguish direct cholecystectomy data from extrapolated recommendations and use the GRADE framework to grade evidence. An overview of the proposed risk-stratified clinical decision algorithm is provided in Figure 1.

## 2 Methods

This is a narrative review, not a systematic review. We conducted a non-systematic literature search of PubMed and Google Scholar for English-language articles published between January 2010 and June 2026, using combinations of the following keywords: “cholecystectomy”, “postoperative nutrition”, “ERAS”, “nutritional support”, “fat malabsorption”, “fat-soluble vitamins”, “immunonutrition”, and “clinical guidelines”. Additional references were identified by manually searching the bibliographies of retrieved articles and by including major guidelines (e.g., ESPEN 2017, ASPEN 2016). We did not apply PRISMA or formal risk-of-bias assessment. The synthesis is descriptive and analytical, focusing on key evidence, controversies, and practical recommendations. We prioritized guidelines, systematic reviews, and RCTs; when direct cholecystectomy evidence was lacking, we explicitly label recommendations as extrapolated (grade C).

## 3 nutritional metabolism and risk assessment after cholecystectomy

### 3.1 Postoperative digestive changes and associated nutritional risks

Cholecystectomy eliminates the bile reservoir, causing continuous bile flow and relative postprandial bile insufficiency (23). This impairs emulsification and absorption of dietary fats and fat-soluble vitamins (A, D, E, K), in a subset of patients, particularly those with persistent symptoms (24). The prevalence of clinically significant fat malabsorption after uncomplicated cholecystectomy is not well established; most patients adapt over time (25). Concurrently, surgical stress increases catabolism, protein breakdown, and insulin resistance, raising protein demands especially in malnourished or critically ill patients (26). Early symptoms like bloating, diarrhea (incidence varies widely, 5-30% depending on definition), and dyspepsia may limit oral intake.

It is crucial to distinguish between:

- Transient postoperative dietary intolerance (resolves within days).
- Post-cholecystectomy diarrhea (often due to bile acid malabsorption).
- Bile acid diarrhea (excess bile acids reaching the colon).
- Post-cholecystectomy syndrome (vague abdominal pain, dyspepsia).
- True steatorrhea (objectively confirmed fecal fat >7 g/day).
- Pancreatic exocrine insufficiency (after severe pancreatitis).

These conditions have different pathophysiologies and require different management. Routine monitoring of fat-soluble vitamin levels is not recommended for all patients; it should be reserved for those with persistent diarrhea, steatorrhea, malnutrition, or specific risk factors (e.g., cholestasis, pancreatic disease). Protein status should be assessed using a combination of weight loss, dietary intake, muscle mass, and inflammatory markers, rather than isolated albumin/prealbumin, which are affected by inflammation and fluid status.

### 3.2 Tools for postoperative nutritional risk identification

Preoperative screening using validated tools (NRS-2002) is recommended for all surgical patients (27). NRS-2002 scores ≥3 indicate need for specialized nutritional support (28). Postoperative reassessment within 24-48 h is reasonable for high-risk patients (age >70, BMI <18.5 or >30, diabetes, preoperative weight loss >5%). For these patients, further assessment may include body composition, handgrip strength, and functional measures (e.g., grip strength). Serum albumin/prealbumin, if used, should be interpreted as prognostic markers, not as direct indicators of nutritional status (29). Note: The NRS-2002 has not been specifically validated in cholecystectomy populations; its use is extrapolated from general surgical guidelines. Common nutrient deficiencies and individualized nutritional management strategies for special populations are summarized in Tables 1 and 2.

**Table 1:** Common nutrient deficiencies.

Nutrient	Deficiency signs/symptoms	Suggested supplementation (oral)	Monitoring	Precautions/Interactions
Vitamin A	Night blindness, dry skin	5,000-10,000 IU/week	Serum retinol at 3-6 months	Teratogenic; avoid in pregnancy; hepatotoxic in high doses
Vitamin D	Bone pain, myopathy	800-2,000 IU/day; higher if severe deficiency	25(OH)D at 3 months	Safe; monitor calcium if high doses
Vitamin E	Neuropathy, ataxia	30-50 IU/day	Serum α-tocopherol	Increases bleeding risk; caution with anticoagulants
Vitamin K	Easy bruising, elevated INR	90-120 µg/day	PT/INR only if anticoagulated	Antagonizes warfarin; adjust dose
Iron	Fatigue, microcytic anemia	65-200 mg elemental iron/day (if deficient)	Ferritin, hemoglobin	GI upset; constipation
Calcium	Bone loss, tetany	500-1,000 mg/day (if low intake)	Serum calcium, PTH	Kidney stones if excessive
Essential fatty acids	Dry scaly rash, poor wound healing	*Linoleic acid (10-15 g/day as safflower oil)*	Clinical assessment	Not routinely needed after cholecystectomy

Routine supplementation for all cholecystectomy patients is not recommended; treat only confirmed deficiency.

**Table 2:** Individualized nutritional management in special populations after cholecystectomy.

Population	Core Strategies	Evidence Level & Limitations	References
Diabetes	Safe glycemic control (140-180 mg/dL); coordinate insulin; protein 1.2-1.5 g/kg	GRADE: Low certainty (indirect)	(32)
Obesity	Caloric control (not restriction); protein 1.5-2.0 g/kg ideal weight; ERAS + mobilization	GRADE: Very low certainty (extrapolated from bariatric)	(33)
Elderly	Nutrient-dense, texture-modified foods; ONS; comprehensive geriatric assessment	GRADE: Low certainty (observational)	(34)

## 4 Implementation of personalized nutrition plans

### 4.1 Phase-Based staged nutritional support

For uncomplicated elective laparoscopic cholecystectomy, early resumption of a normal diet as tolerated is recommended by ERAS protocols (30). However, a phased approach may be helpful for patients with nausea, vomiting, or ileus. The following phases are suggested, but advancement should be based on individual tolerance, not mandatory timelines:

- Phase 1 (0–24 h): Clear liquids (water, broth) can be started within 6-8 h. Evidence from colorectal surgery suggests this reduces ileus and hospital stay (31). (GRADE: moderate certainty, strong recommendation, indirect evidence for cholecystectomy).
- Phase 2 (days 1–3): Low-fat, low-fiber soft foods (e.g., porridge, noodles, steamed egg custard).
- Phase 3 (day 4 to several weeks): Gradual reintroduction of normal foods, avoiding high-fat meals if intolerant. Protein intake 1.2-1.5 g/kg/day for patients at risk of malnutrition; for well-nourished patients, 0.8-1.0 g/kg/day may suffice.

#### 4.1.1 Controversy

There is no direct RCT evidence that a staged low-fat diet is superior to an unrestricted diet as tolerated in uncomplicated laparoscopic cholecystectomy. The phased approach is clinically pragmatic but not strongly evidence-based.

#### 4.1.2 Actionable insight

Use a “feeding tolerance checklist” (distension, pain, diarrhea, nausea) before each phase transition. This checklist is author-proposed and not validated.

### 4.2 Optimizing specific nutrients in postoperative care

Specific nutrient recommendations (with GRADE evidence):

- Fat: For patients with documented fat malabsorption or steatorrhea, consider replacing long-chain triglycerides (LCTs) with MCT oil (10-20 g/day). MCTs do not provide essential fatty acids and should not completely replace LCTs. Evidence: GRADE low certainty, weak recommendation (small RCTs, *n* = 45, indirect). Potential adverse effects: abdominal cramping, diarrhea.
- Fat-soluble vitamins: Do not routinely screen all patients. Screen only those with persistent diarrhea (>4 weeks), steatorrhea, weight loss, cholestasis, or pancreatic insufficiency. If deficient: supplement per guidelines (e.g., vitamin D 800-2000 IU/day maintenance; higher for deficiency). Evidence: GRADE very low certainty, weak recommendation (extrapolated from malabsorption syndromes). Vitamin A supplementation is teratogenic and hepatotoxic; vitamin E may increase bleeding risk; vitamin K interacts with warfarin. Always assess contraindications.
- Fiber: For diarrhea → soluble fiber (psyllium, 5-10 g/day). For constipation → insoluble fiber plus hydration.
- Probiotics: Not routinely recommended. Some small RCTs in mixed surgical populations show benefit for bloating, but strain-specific effects and heterogeneity limit generalizability. *Evidence: GRADE low certainty, weak against recommendation.* Use only in research settings or after careful consideration of risks (immunocompromised, severe illness).
- Protein: 1.2-1.5 g/kg actual body weight for malnourished or high-risk patients; for obese patients, use ideal body weight; for those with renal impairment, adjust downward. BCAAs (leucine 2-3 g/day) may be considered in sarcopenic patients.

## 5 Nutritional care in high-risk groups and outcome evaluation

### 5.1 Nutritional management in patients with metabolic comorbidities

#### 5.1.1 Diabetes mellitus

Safe glycemic control (not overly tight) is important. Perioperative glucose targets: 140-180 mg/dL for most patients. Coordinate with insulin or oral agents; avoid hypoglycemia. Low-glycemic index carbohydrates may help, but the priority is adequate intake to support healing (32). Protein 1.2-1.5 g/kg/day. Evidence: GRADE low certainty (indirect from diabetic surgery guidelines).

#### 5.1.2 Obesity (BMI >30)

Avoid both overfeeding and excessive restriction. Energy intake should meet healing needs (∼25-30 kcal/kg ideal weight). Protein 1.5-2.0 g/kg ideal body weight; monitor for sarcopenic obesity. These recommendations are extrapolated from bariatric surgery; no cholecystectomy-specific trials (33). Evidence: GRADE very low certainty, weak recommendation.

#### 5.1.3 Elderly (≥70 y)

Assess frailty, swallowing, dentition, cognition, social support. Use GNRI or other validated tools; GNRI ≤92 is one predictor but not validated specifically for cholecystectomy. Provide nutrient-dense, texture-modified foods; oral nutritional supplements (ONS) with protein, vitamin D, calcium (34). Evidence: GRADE low certainty (observational studies).

### 5.2 Evaluation of nutritional care protocols

Future studies should use patient-centered outcomes such as:

- Primary:Time to tolerate normal diet; GI symptom scores (e.g., PROMIS GI); unplanned healthcare contacts.
- Secondary: Energy/protein intake, weight change, quality of life.
- Long-term: Metabolic syndrome, bone density (only in high-risk cohorts) at 1-2 years.

Albumin and prealbumin are not recommended as primary nutritional outcomes due to confounding by inflammation.

## 6 Standardized protocols and Complex scenarios

### 6.1 Standardized postoperative nutrition protocols in elective cholecystectomy

For elective laparoscopic cholecystectomy, ERAS protocols (e.g., preoperative carbohydrate loading, early oral feeding) are well established and supported by the ESPEN 2017 guideline (12). Key ESPEN recommendations:

- Avoid prolonged fasting; clear fluids up to 2 h pre-op.
- Early oral feeding within 24 h is safe.
- Routine parenteral nutrition not indicated if oral intake >60%.
- Nutritional risk screening (NRS-2002) should be performed.

Our phase-based recommendations align with ESPEN but expand on cholecystectomy-specific issues (fat malabsorption, MCT, vitamins) with appropriate caution.

## 7 Special considerations in complicated scenario

### 7.1 Acute cholecystitis

- Pathophysiology: Inflammation, possible sepsis, catabolism.
- Nutritional risks: Protein catabolism up to 2.0 g/kg/day in severe cases (extrapolated from critical illness guidelines). Risk of refeeding syndrome after prolonged fasting.

Recommendations:

- Conservative management without immediate surgery: enteral nutrition (nasogastric) if oral intake <60% for >48 h and gastrointestinal function is intact (based on ASPEN 2016).
- After cholecystectomy: same phased approach but monitor for ileus; advance more slowly.
- Evidence: No RCTs specifically for acute cholecystitis; extrapolated from acute pancreatitis guidelines.

### 7.2 Post-pancreatitis or cholangitis

Pancreatic exocrine insufficiency (PEI) occurs only after severe pancreatitis with necrosis, not after uncomplicated biliary pancreatitis. Do not assume PEI in all cases.

Recommendations:

- If PEI is documented (fecal elastase <200 µg/g, or steatorrhea with weight loss), start pancreatic enzyme replacement therapy (25,000-50,000 lipase units per meal, titrated to symptoms).
- Fat intake should be tolerated according to patient response; no arbitrary <30 g/day restriction unless symptoms dictate.
- Monitor fat-soluble vitamins every 3-6 months for the first year if PEI is confirmed.
- Evidence: GRADE low certainty (extrapolated from pancreatitis guidelines).

### 7.3 Emergency vs. Elective Surgery

- Emergency cholecystectomy carries higher nutritional risk due to preoperative fasting, sepsis, and catabolism. Perform early nutritional assessment (NRS-2002) within 24 h; start enteral or parenteral support if indicated.

## 8 Future research directions

Based on identified evidence gaps, we propose concrete priorities:

1. RCT comparing MCT-enriched diet vs. standard low-fat diet in patients with post-cholecystectomy diarrhea (primary: GI symptom score at 1 month).
2. Pragmatic cluster RCT comparing routine vitamin screening vs. no screening on 6-month bone mineral density in elderly high-risk patients.
3. Patient-reported outcomes (GIQLI, PROMIS GI) in future trials.
4. Prehabilitation (nutrition + exercise) in frail elderly before elective cholecystectomy.
5. Cost-effectiveness of universal vitamin D supplementation vs. targeted screening.

## 9 Conclusion

Postoperative nutritional care after cholecystectomy has evolved from fasting to ERAS-based protocols. However, direct high-quality evidence specific to cholecystectomy is scarce. This narrative review provides a critical synthesis, emphasizing that:

1. Routine NRS-2002 screening identifies at-risk patients.
2. Early oral feeding is safe and beneficial (indirect evidence).
3. MCT oil and vitamin monitoring should be reserved for symptomatic/high-risk patients.
4. Special populations and complicated scenarios require tailored strategies, but most recommendations are extrapolated.

We caution against routine supplementation or testing. Multidisciplinary collaboration and digital health tools offer promise. Future RCTs are urgently needed to validate these recommendations. This framework aims to guide clinicians while acknowledging substantial evidence gaps.
